# PD-1 N58-Glycosylation-Dependent Binding of Monoclonal Antibody Cemiplimab for Immune Checkpoint Therapy

**DOI:** 10.3389/fimmu.2022.826045

**Published:** 2022-03-02

**Authors:** Dan Lu, Zepeng Xu, Ding Zhang, Min Jiang, Kefang Liu, Juanhua He, Dongli Ma, Xiaopeng Ma, Shuguang Tan, George F. Gao, Yan Chai

**Affiliations:** ^1^ Savaid Medical School, University of Chinese Academy of Sciences, Beijing, China; ^2^ CAS Key Laboratory of Pathogenic Microbiology and Immunology, Institute of Microbiology, Chinese Academy of Sciences, Beijing, China; ^3^ Faculty of Health Sciences, University of Macau, Macau, Macau SAR, China; ^4^ Shanxi Academy of Advanced Research and Innovation, Taiyuan, China; ^5^ College of Life Sciences, Jiangxi Science and Technology Normal University, Nanchang, China; ^6^ Shenzhen Children’s Hospital, Shenzhen, China

**Keywords:** PD-1, antibody, N58 glycosylation, cemiplimab, immune checkpoint therapy (ICT)

## Abstract

Immune checkpoint therapy (ICT) with a monoclonal antibody (MAb) against programmed cell death protein 1 (PD-1) is a powerful clinical treatment for tumors. Cemiplimab is a human IgG4 antibody approved in 2018 and is the first MAb proven to be effective for locally advanced basal cell carcinoma. Here, we report the crystal structure of cemiplimab bound to PD-1 and the effects of PD-1 N-glycosylation on the interactions with cemiplimab. The structure of the cemiplimab/PD-1 complex shows that cemiplimab mainly binds to PD-1 with its heavy chain, whereas the light chain serves as the predominant region to compete with the binding of PD-L1 to PD-1. The interaction network of cemiplimab to PD-1 resembles that of camrelizumab (another PD-1-binding MAb), and the N58 glycan on the BC loop of PD-1 may be involved in the interaction with cemiplimab. The binding affinity of cemiplimab with PD-1 was substantially decreased with N58-glycan-deficient PD-1, whereas the PD-1/PD-L1 blocking efficiency of cemiplimab was attenuated upon binding to the N58-glycosylation-deficient PD-1. These results indicate that both the binding and blocking efficacy of cemiplimab require the N58 glycosylation of PD-1. Taken together, these findings expand our understanding of the significance of PD-1 glycosylation in the interaction with cemiplimab.

## Introduction

Immune checkpoint therapy (ICT), also called immune checkpoint blockade (ICB), has been widely used in tumor immunotherapy since the approval of the CTLA-4-specific ipilimumab in 2011 (1–3). Encouragingly, the blocking of the programmed cell death protein-1 (PD-1)/PD-1 ligand 1 (PD-L1) pathway with monoclonal antibodies (MAbs) has dramatically improved the treatment prospects for multiple tumors (2, 4). PD-1 belongs to the immunoglobulin gene superfamily, which was identified in T cells upon programmed cell death (5). PD-1 is mainly expressed in immune cells, including activated dendritic cells, natural killer cells, T cells, and B cells (6). The ligand for PD-1, PD-L1, is upregulated in a broad range of tumor cells and mediates tumor immune escape through interaction with PD-1 (7). Inhibition of the PD-1/PD-L1 interaction with MAbs restores T-cell function to retain preexisting antitumor activity (4, 8). Currently, there are 10 clinically approved anti-PD-1 MAbs: nivolumab (Bristol-Myers Squibb, 2014), pembrolizumab (Merck Sharp & Dohme, 2014), cemiplimab (Sanofi and Regeneron, 2018), toripalimab (Junshi, 2018), sintilimab (Innovent, 2018), camrelizumab (HengRui, 2019), tislelizumab (BeiGene, 2019), dostarlimab (Tesaro, 2021), penpulimab (Chia Tai-Tianqing, 2021), and zimberelimab (Gloria Biosciences, Arcus Biosciences, and Taiho, 2021).

Glycosylation is a common protein post-translational modification, and it plays critical roles in multiple biological processes (9). For instance, N-glycosylation is important in the maintenance of the surface expression of PD-1 protein and the regulation of the interaction with PD-L1 (10). Our previous work demonstrates that the extracellular domain of PD-1 (PD-1-ECD) is extensively glycosylated, and N-glycosylation is found in all the four potential N-glycosylation sites (N49, N58, N74, and N116) (11, 12).

Structural evidence suggests the impact of N-glycans on PD-1 interaction with MAbs. Among the four N-glycosylation sites, structural evidence suggests that N58 in the BC loop of PD-1 is located near the interface between PD-1 and PD-L1, whereas glycosylation at N58 is not involved in the binding of PD-1 to PD-L1 (11, 13). The reported complex structures of PD-1 with nivolumab, pembrolizumab, tislelizumab, or toripalimab show that PD-1 glycosylation does not engage with these MAbs (11, 14–16). In contrast, PD-1 N-glycans are involved in the binding to some anti-PD1 antibodies (11, 17). We previously reported that PD-1 glycosylation at N58 promotes the interaction with camrelizumab, and the blocking efficacy of camrelizumab is dampened upon binding to N58 glycosylation-deficient PD-1 (17). Furthermore, the binding of other PD-1-specific MAbs (e.g., MW11-h317, mAb059c, and STM418) also involves the glycans at the N58 site (12, 18, 19). Structural evidence shows that these MAbs mainly engage with the conserved core region of the glycan chains at N58.

Cemiplimab (REGN2810, Libtayo^®^), co-developed by Sanofi and Regeneron, is a fully human IgG4 MAb specific to the PD-1 receptor (18). It was approved by the US Food and Drug Administration (FDA) for clinical treatment of metastatic cutaneous squamous cell carcinoma (CSCC) in 2018 (19, 20). Cemiplimab is the first MAb proven to be effective for locally advanced basal cell carcinoma, a tumor with no standard treatment regimen after first-line hedgehog inhibitor therapy (21). Here, we report the molecular basis of cemiplimab binding to PD-1 through the determination of the cemiplimab/PD-1 complex structure, and we investigated the roles of PD-1 N-glycosylation in the cemiplimab interaction. We found that both the binding and inhibition efficacies of cemiplimab to PD-1 were promoted by PD-1 N58 glycosylation. The findings observed in the present study expand our knowledge of the interaction mechanisms of glycosylation for antibodies to PD-1 in the context of tumor ICT.

## Results

### Overall Structure of Cemiplimab/PD-1 Complex

**Figure 1:**
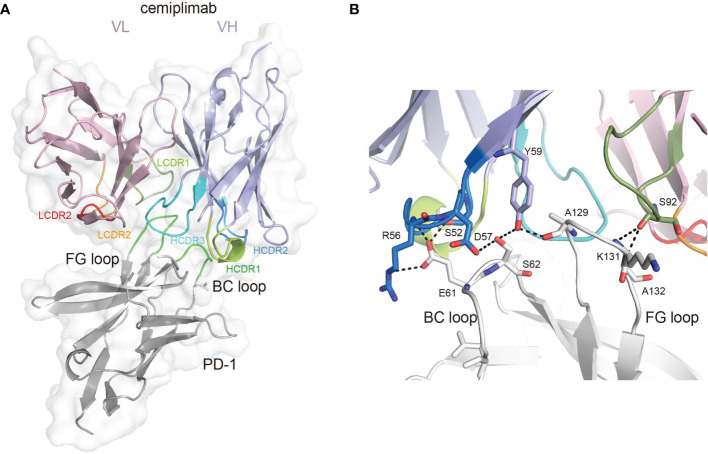
The binding mechanism of cemiplimab to PD-1. **(A)** Overall structure of cemiplimab bound to PD-1. PD-1 colored in gray is shown as surface representation, while the heavy chain (VH) and light chain (VL) of cemiplimab-scFv are shown as cartoon colored in light blue and light pink. The CDR1, CDR2, and CDR3 loops of the heavy chain are colored in green (HCDR1), blue (HCDR2), and cyan (HCDR3), respectively. The CDR1, CDR2, and CDR3 loops of the light chain are colored in limon (LCDR1), orange (LCDR2), and red (LCDR3), respectively. The BC and FG loops are colored in green. **(B)** The detailed binding of cemiplimab to the FG and BC loops of PD-1. The residues taking part in forming hydrogen bonds are shown as sticks. The hydrogen bonds between residues are shown as a dashed line in black.

To investigate the binding mechanisms of cemiplimab to PD-1, the PD-1-ECD (PD-1-*E. coli*) protein and single-chain variable fragment (scFv) of cemiplimab were expressed in *Escherichia coli* cells as inclusion bodies and renatured by *in vitro* refolding (11, 17). The cemiplimab-scFv/PD-1 complex was prepared after *in vitro* co-refolding and used for crystal screening (**Supplementary Figure 1**). Diffractable crystals were obtained, and the structure of the cemiplimab-scFv/PD-1 complex was solved at a resolution of 3.4 Å, with Rwork and Rfree values of 0.245 and 0.285, respectively (**Table 1**). The overall structure reveals that PD-1-ECD and cemiplimab-scFv form a 1:1 complex, and the interaction of cemiplimab with PD-1 buries a total surface area of 1,614 Å. Cemiplimab binds to PD-1 with all three complementarity-determining regions (CDRs) from its heavy chain and the LCDR3 from the light chain variable domain (VL) (**Figure 1A** and **Table 2**). Specifically, there are multiple hydrogen bond interactions between residues from LCDR3 (S92) of cemiplimab and the FG loop of PD-1 (K131 and A132) and between residues from HCDR2 (S52, R56, D57, and Y59) of cemiplimab and the BC loop (E61 and S62) and FG loop (A129) of PD-1 (**Figure 1B** and **Table 2**; **Supplementary Figure 2**).

**Table 1:** Crystallographic data collection and refinement statistics.

	Cemiplimab/PD-1
**Data collection**	
Space group	*P 3_2_ 2 1*
Wavelength (Å)	0.97853
Unit cell dimensions	
a, b, c (Å)	131.54, 131.54, 124.34
α, β, γ (°)	90.00, 90.00, 120.00
Resolution (Å)	50.00-3.40 (3.58-3.40)*
Unique. reflections	17,566
*R_merge_*	0.242 (0.658)
I/σ	9.50 (4.0)
Completeness (%)	100.0 (100.0)
Redundancy	8.9 (8.5)
**Refinement**	
*R_work_*/*R_free_*	0.247/0.287
No. atoms	
Protein	5176
Ligands	0
Water	0
**RMS deviations**	
Bond lengths (Å)	0.003
Bond angles (°)	0.690
**Ramachandran plot**	
Favored (%)	94.95
Allowed (%)	5.05
Outliers (%)	0.00

*Values in parentheses are for highest-resolution shell.

**Table 2:** Residues contributed interaction between cemiplimab and PD-1.

Cemiplimab	PD-1	Contacts^a^	Total
**H chain**			145
**T28**	R86	1	
**N31**	F82, P83	2, 5	
**F32**	P83, E84, D85	7, 4, 2	
**S52**	E61, S62	9 (1)^b^, 2	
**G54**	E61	10	
**G55**	E61	4	
**R56**	E61	20 (2)	
**D57**	S60, E61, S62	7, 7, 6 (1)	
**Y59**	S62, A129, P130	6 (1), 20 (1), 4	
**K98**	D85	1	
**W99**	L128	3	
**G100**	V64, P83, L128	2, 1, 2	
**N101**	V64, K78	6, 1	
	I126, L128	1, 4	
**I102**	I126, L128	3, 2	
**Y103**	K78, D85	1, 1	
**D105**	D85	1	
**L chain**			50
**F32**	I126, A132, Q133	1, 14, 2	
**Y49**	K78	2	
**S91**	A132	2	
**S92**	P130, K131, A132	1, 5 (1), 7 (1)	
**N93**	K131	7	
**T94**	A129, P130	2, 7	

a Numbers represent the number of atom-to-atom contacts between cemiplimab and PD-1 residues, which were analyzed by the Contact program in CCP4 suite (the distance cutoff is 4.5 Å).

b Numbers in the parentheses represent the number of hydrogen bonds between cemiplimab and PD-1 residues, which were analyzed by the Contact program in CCP4 suite (the distance cutoff is 3.5 Å).

**Figure 2:**
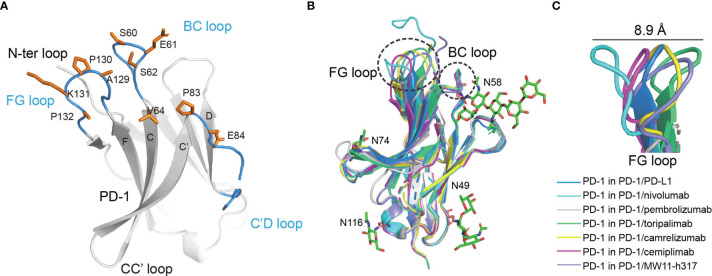
The structure characterization of PD-1 upon binding to MAbs. **(A)** The key region of PD-1 binding to cemiplimab. The β-strands of PD-1 are represented as the capital characters C, C′, D, F, and G, respectively. The CC′, C′D, and FG loops of PD-1 are highlighted in the blue cartoon, and the key epitopes of PD-1 binding to cemiplimab are shown as orange sticks, respectively. **(B)** Superposition of PD-1 upon binding to the PD-L1 ligand or different MAbs, including the PD-1 extracted from the complex structures of PD-1/PD-L1 (blue) (PDB code: 4ZQK), PD-1/nivolumab (cyan) (PDB code: 5WT9), PD-1/pembrolizumab (gray) (PDB code: 5JXE), PD-1/toripalimab (green) (PDB code: 6JBT), PD-1/camrelizumab (yellow) (PDB code: 7CU5), PD-1/cemiplimab (magenta) and PD-1/MW11-h317 (light blue) (PDB code: 6JJP). FG loop and BC loop of PD-1 which contributed vital binding to the cemiplimab are shown as a dashed line in black. **(C)** Flexible conformations of the FG loop of PD-1 upon binding to PD-L1 or different MAbs.

Cemiplimab mainly binds to the BC and FG loops of PD-1 through its HCDR2, HCDR3, and LCDR3 loops (**Figure 2A**). Of note, the HCDR2 (G53 and G54) of cemiplimab is close to N58 of PD-1. The structure of PD-1 molecules with PD-L1, nivolumab, pembrolizumab, toripalimab, camrelizumab, and MW11-h317 were then superimposed with that from the cemiplimab/PD-1 complex to investigate the conformational changes upon binding to different MAbs (**Figure 2B**). Pembrolizumab predominantly binds to the C’D loop of PD-1, toripalimab mainly binds to the FG loop, and the binding of nivolumab is mainly located on the N-terminal loop of PD-1. In contrast, camrelizumab, MW11-h317, and cemiplimab mainly bind to the BC and FG loops, while camrelizumab and MW11-h317 contact the N58 glycan chains at the BC loop of PD-1. The FG loop exhibits substantial conformational variation when bound to different MAbs, while the BC loop shows limited conformational changes (**Figure 2B**). Of note, the FG loop of PD-1 in the cemiplimab/PD-1 complex exhibits a similar conformation to that in the PD-1/PD-L1 complex, while varied conformational changes were induced upon binding to the other MAbs (**Figure 2C**).

### PD-1/PD-L1 Blocking Mechanisms by Cemiplimab

**Figure 3:**
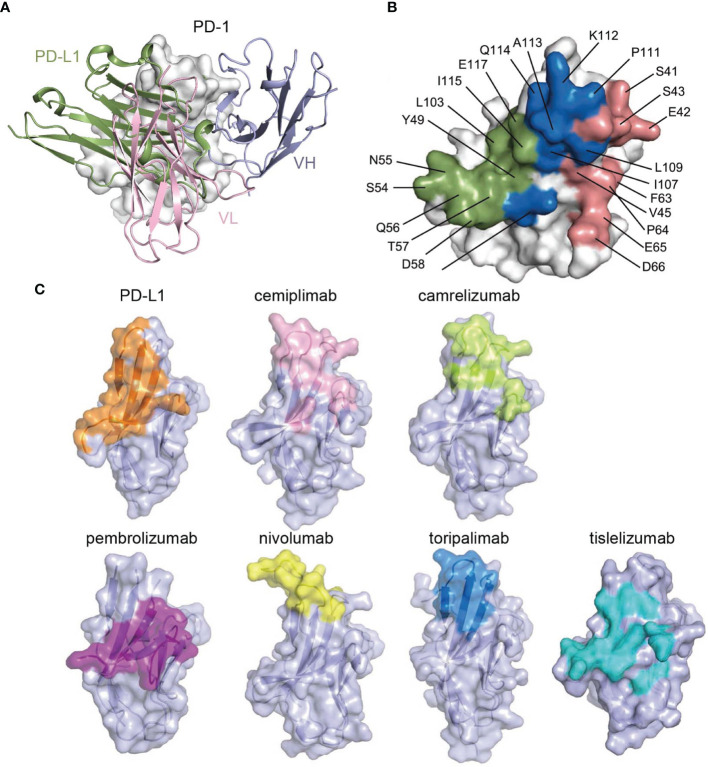
Structural basis of the blockade binding of cemiplimab with PD-L1. **(A)** Comparison of cemiplimab/PD-1 with PD-L1 extracted from PD-1/PD-L1 complex structure (PDB code: 4ZQK). PD-L1 is shown as smudge cartoon, while PD-1 is shown as surface diagram in white. VH and VL of cemiplimab-scFv are shown as cartoons in light blue and light pink, respectively. **(B)** The competitive binding surfaces of cemiplimab with PD-L1 on PD-1. The residues bound to cemiplimab alone are colored in deep salmon, while the residues contact with PD-L1 alone are colored in smudge, and the residues contacted by both cemiplimab and PD-L1 are colored in blue. The epitope residues in PD-1 are pointed out in black characters. **(C)** The binding surface of PD-L1 and structurally known clinically approved MAbs on PD-1 is shown in different colors. The binding surface of PD-L1 and other MAbs, e.g., cemiplimab, camrelizumab, pembrolizumab, nivolumab, toripalimab, and tislelizumab are colored in orange, light pink, limon, purple, yellow, blue, and teal, respectively.

The structure of the cemiplimab/PD-1 complex was next superimposed with that of the PD-1/PD-L1 complex (PDB: 4ZQK) to analyze the PD-1/PD-L1 inhibition mechanism of cemiplimab. The analysis revealed that the major domain of cemiplimab responsible for inducing stereospecific hindrance to the binding of PD-L1 is the VL domain (**Figure 3A**). Additionally, the binding area of cemiplimab on PD-1 substantially overlaps with that of PD-L1 (**Figure 3B**). Together with the superior binding affinity of cemiplimab over PD-L1 to PD-1, the binding of cemiplimab would abrogate PD-L1 binding to PD-1 and inhibit PD-L1-mediated signaling. Among the clinically approved MAbs, the binding area of cemiplimab is similar to that of toripalimab, camrelizumab, nivolumab, and MAbs engaging mainly with the FG loop of PD-1 (e.g., MW11-h317, mAb059c, and GY5), whereas it is distinct from that of pembrolizumab and tislelizumab (**Figure 3C** and **Supplementary Figure S2**). Taken together, our structural analyses indicate that cemiplimab blocks the PD-1/PD-L1 interaction mainly through its VL domain.

### Structural Indications for N58 Glycosylation of PD-1 to Interact With Cemiplimab

**Figure 4:**
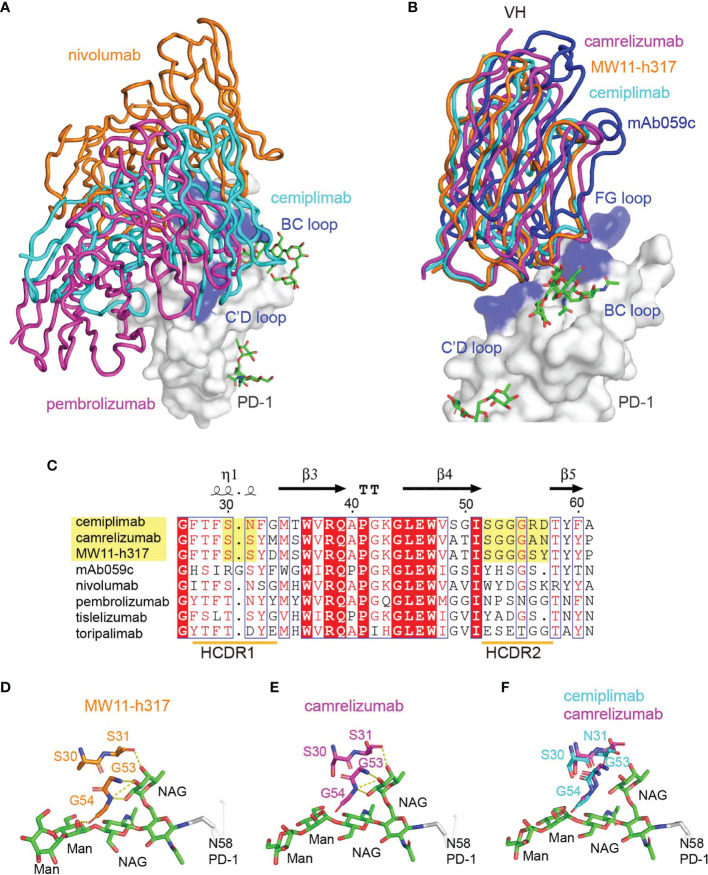
Interaction between MAbs and N58 glycosylation on BC loop. **(A)** The comparison of the overall binding of cemiplimab, nivolumab, and pembrolizumab to PD-1. Superimposition of cemiplimab/PD-1 complex with that of pembrolizumab/PD-1 (PDB: 5JXE) and nivolumab/PD-1 (PDB: 5WT9). The cemiplimab, pembrolizumab, and nivolumab are shown as ribbon and colored in cyan, magenta, and orange, respectively. PD-1 extracted from camrelizumab/PD-1 (PDB code: 7CU5) complex is shown as surface representation colored in white. **(B)** Superimposition of cemiplimab/PD-1 complex with that of camrelizumab (PDB: 7CU5), MW11-h317 (PDB: 6JJP) and mAb059c (PDB: 6K0Y). The VH domains of the MAbs are shown as ribbons while the VL domains are not shown. PD-1, cemiplimab, camrelizumab, MW11-h317, and mAb059c are colored in gray, cyan, magenta, orange and blue, respectively. The CC′, C′D, and FG loops of PD-1, which participate in binding to Mabs are highlighted in blue. **(C)** Structure-based sequence alignment of cemiplimab and other anti-PD-1 MAbs. Coils above the sequences indicate α-helices, and the lines with arrowhead represent the β sheets. Residues highlighted in yellow are highly conserved. The sequence alignment was generated with ClustalX and ESPript. **(D, E)** The interaction of N-glycosylation N58 with MW11-h317 **(D)** or camrelizumab **(E)**. The amino acid residues involved in hydrogen bond interaction and N58 glycans are shown as sticks, with amino acids in MW11-h317 colored in orange, camrelizumab colored in magenta, and the glycans in PD-1 colored in green. Hydrogen bonds are labeled by yellow dashed lines. **(F)** Comparison of cemiplimab/PD-1 complex with that of camrelizumab (PDB: 7CU5), and the amino acids in cemiplimab are colored in cyan and residues in camrelizumab are colored in magenta.

To compare the binding mode of PD-1-targeting MAbs, the structure of the cemiplimab/PD-1 complex was then superimposed with the nivolumab/PD-1 and pembrolizumab/PD-1 complexes, with the structure of PD-1 fixed. These comparative analyses show that nivolumab and pembrolizumab adopt distinct binding orientations compared to cemiplimab, although the binding surface with nivolumab highly overlaps (**Figure 4A**). Comparative analysis with other N58 glycan-engaged MAbs revealed that the orientation of cemiplimab upon binding to PD-1 resembles that of camrelizumab and MW11-h317, while the binding of mAb059c is substantially biased toward the FG-loop (**Figure 4B**). Alignment of these MAbs’ sequences reveals that the heavy chains of the glycosylation-engaged MAbs camrelizumab, MW11-h317, and cemiplimab share similar HCDR2s compared to those from other glycosylation-independent MAbs, except for mAb059c that also contacts the N-glycan at N58 (**Figure 4C**). In the MW11-h317/PD-1 and camrelizumab/PD-1 complex structures, the N-acetylglucosamine (NAG) and mannose (MAN) form multiple hydrogen bond interactions with residues from HCDR1 (S31) and HCDR2 (G53 and G54) (**Figures 4D, E**). Although the *E. coli-*expressed PD-1-ECD proteins used in this study for crystal growing did not contain any glycan modification, the conserved conformation of the HCDR1 and HCDR2 of cemiplimab with that of camrelizumab indicates that the glycan chains of PD-1 N58 would form a similar interaction network through amino acids from HCDR1 and HCDR2 of cemiplimab (**Figure 4F**).

### The N58 Glycan of PD-1 Promotes the Binding and Blocking Efficacy of Cemiplimab

**Figure 5:**
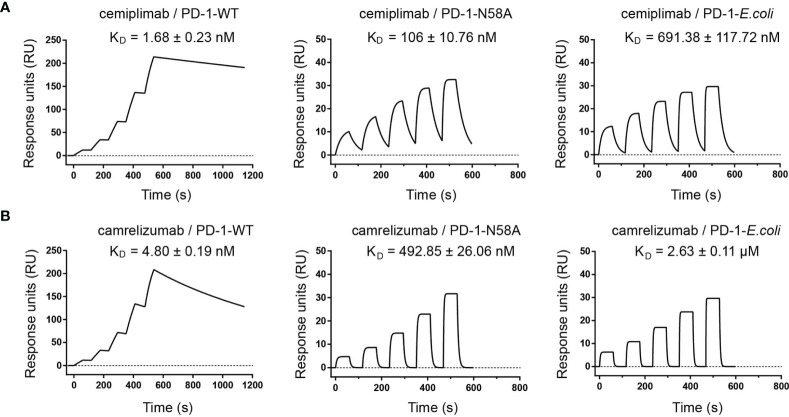
N-glycosylation of N58 remotes the binding to cemiplimab. **(A)** SPR assay characterization of the binding profiles of cemiplimab with PD-1-WT (left), PD-1-N58A (middle) proteins expressed in 293F cells, and PD-1-*E. coli* (right) expressed in *E. coli* cells. **(B)** SPR assay characterization of the binding of camrelizumab with PD-1-WT (left), PD-1-N58A (middle) expressed in 293F cells, and PD-1-*E. coli* (right) expressed in *E. coli* cells. The mean value of the KD was recorded after repeating each experiment three times.

Based on this structural information, we speculated that the N58 glycan of PD-1 potentially plays a role in binding to cemiplimab, although the N58 glycan chains were not observed with the PD-1-*E. coli* proteins used in the structural study. Therefore, we further evaluated the binding profiles of cemiplimab with wild type (WT) PD-1 protein (PD-1-WT) expressed in 293F cells, which enabled full glycosylation on proteins similarly to host cells using surface plasmon resonance (SPR). Additionally, N58A-mutated PD-1 protein (PD-1-N58A) expressed in 293F cells, which is specifically deficient in N58 glycosylation, was also investigated. The binding affinity (evaluated as K_D_) of cemiplimab to N58-mutated PD-1 protein substantially decreased to 106 nM, a 60-fold reduction compared to PD-1-WT (K_D_ = 1.68 nM) (**Figure 5A**). Moreover, we analyzed the binding profiles of cemiplimab to PD-1-ECD proteins obtained from *E. coli* cells, which carried no post-translational modifications. The SPR analysis revealed a similar reduction in binding affinity to PD-1 protein from *E. coli* (K_D_ = 691.38 nM) compared to that from 293F cells (K_D_ = 1.68 nM) (**Figure 5A** and **Supplementary Table S1**). The binding of camrelizumab, which is promoted by glycosylation of PD-1 N58, was tested in parallel as a control (**Figure 5B**). The decreased binding affinities of camrelizumab with N58A-mutated PD-1 proteins from 293F cells (K_D_ = 492.85 nM) was similar to that of cemiplimab, and the binding affinity of camrelizumab with non-glycosylated PD-1 proteins from *E. coli* (K_D_ = 2.63 μM) is substantially decreased compared with glycosylated PD-1 (K_D_ = 4.8 nM) like cemiplimab (**Figure 5B**). Based on these findings, we concluded that N-glycosylation at N58 promotes the binding of cemiplimab to PD-1.

**Figure 6:**
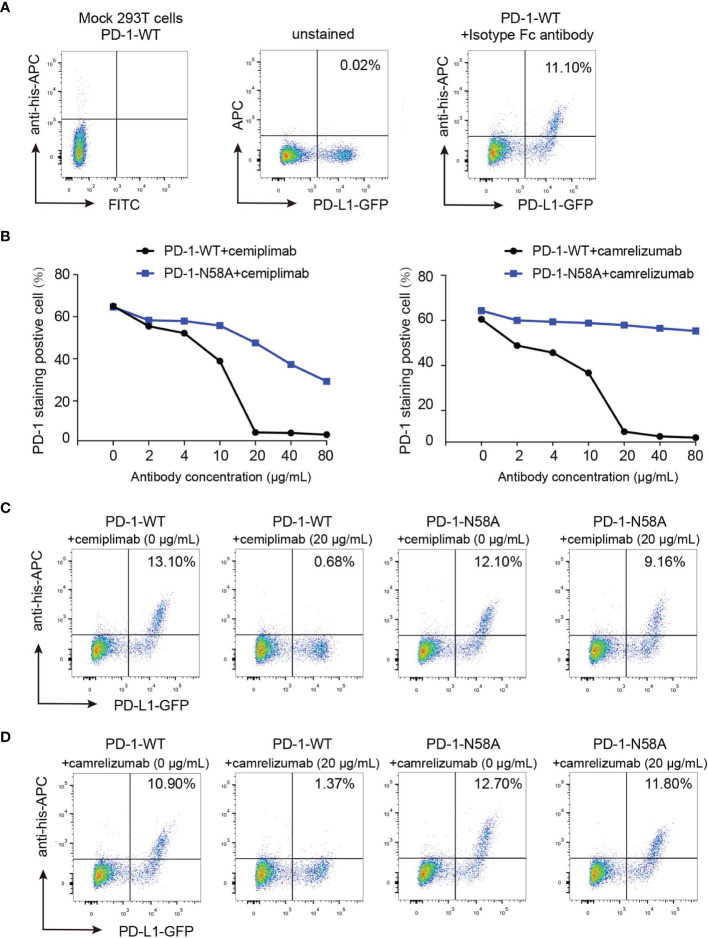
Reduced blocking efficiency of cemiplimab to N58 glycosylation-deficient PD-1. **(A)** Untransfected 293T cells and transfected 293T cells incubated with isotype antibody as negative control. **(B)** The blocking of the binding of His-tagged PD-1-WT (blank) or PD-1-N58A (blue) proteins to PD-L1 expressing 293T cells is analyzed with varying concentrations (0, 2, 4, 10, 20, 40, and 80 μg/ml) of full-length cemiplimab (left) or camrelizumab (right). The PD-L1 expressing 293T cells staining with His-tagged PD-1-WT or PD-1-N58A are prepared as a positive control. **(C, D)** The frequencies of the His-tagged PD-1-WT or PD-1-N58A protein staining positive subpopulations in the absence (0 μg/ml) or presence (20 μg/ml) of cemiplimab **(C)** or camrelizumab **(D)**. At the same concentration (20 μg/ml) of cemiplimab or camrelizumab, the frequency of His-tagged PD-1-WT or PD-1-N58A staining positive cells was calculated based on PD-L1-GFP-positive cells. The experiment was repeated twice in the results averaged.

To verify the roles of N58 glycosylation in PD-1/PD-L1 blocking by cemiplimab, a mechanism believed to be the key aspect for the restoration of antitumor efficacy for MAb-based ICT, we further tested the blocking efficiency of the full-length cemiplimab to N58 glycosylation-deficient PD-1. His-tagged PD-1-WT and PD-1-N58A recombinant proteins were prepared from 293F cells and were used to stain 293T cells transiently expressing PD-L1. The blocking of the PD-1/PD-L1 interaction was analyzed by staining the PD-L1-expressing 293T cells with a mixture of serial dilutions of the full-length cemiplimab or camrelizumab proteins pre-incubated with the same concentrations of WT or N58A-mutated PD-1-His proteins (2 μg/ml). As controls, mock-transfected 293T cells stained with PD-1-WT and PD-L1-transfected 293T cells stained with isotype antibody were enrolled as controls (**Figure 6A**). We found that the frequency of protein-staining-positive cells with PD-1-WT-His protein substantially decreased from 65.0% to 5.0% in the presence of 20 μg/ml cemiplimab, indicating the complete blockade of the PD-1/PD-L1 interaction (**Figures 6B, C**). However, the blocking efficacy of cemiplimab to PD-1-N58A mutant protein with PD-L1 was decreased compared to that of PD-1-WT. No substantial blocking efficacy was observed for cemiplimab with N58A mutant protein, even at the high concentration of 80 μg/ml (**Figure 6B**). The decreased blocking scenario of camrelizumab to N58A mutant PD-1 is similar to that of cemiplimab (**Figures 6B, D**). These results indicate that N58 glycosylation promotes both the binding and blocking of cemiplimab.

## Discussion

In this study, we report the interaction mechanisms between cemiplimab and PD-1. Overall, the binding of cemiplimab to PD-1 resembles that of camrelizumab, as we reported earlier (17). Structural analysis indicates that cemiplimab competes with the binding of PD-L1 to PD-1 with overlapping binding surface areas of PD-L1 resulting in steric hindrance. Cemiplimab mainly binds to the BC and FG loops of PD-1, whereas the FG loop of PD-1 contributes to multiple interactions with PD-L1 and therefore mediates the competitive interaction with cemiplimab or PD-L1 (22). Comparative structural analyses with other solved MAb/PD-1 complex structures suggest that the binding of cemiplimab resembles that of camrelizumab and may be promoted by the glycan chains at N58 on the BC loop. Subsequent analyses indicated that the deficiency of N58 glycosylation substantially decreases both the PD-1 binding affinity and blocking of cemiplimab, which is similar to that of camrelizumab.

The previously reported complex structures of clinically approved MAbs (e.g., nivolumab, pembrolizumab, camrelizumab, toripalimab, and tislelizumab) reveal that these MAbs predominantly bind to the surface or terminal loops of PD-1, whereas PD-L1 mainly binds to the surface on PD-1 constituted by β sheets. Comparative analysis revealed that the flexible surface or terminal loops of PD-1 exhibit distinct conformations upon binding to varied MAbs. The FG loop of PD-1 engages with PD-L1 (22, 23) and is a hot spot loop for PD-1 specific therapeutic MAbs, e.g., toripalimab, camrelizumab, GY-5, and GY-14 (15, 17, 24). Structural analysis revealed that the binding region of cemiplimab on PD-1 is similar to that of camrelizumab, which mainly binds to the BC loop, FG loop, and C′D loop.

Glycosylation is involved in fundamental biological processes and plays pivotal roles in tumor development and progression, immune modulation, and metastasis (25). PD-1 protein is not only upregulated in T cells to mediate immune suppression but is also expressed across a broad range of tumor cells to promote tumor suppression (26). Dysregulated protein glycosylation occurs in tumor cells and tumor-associated dysregulated glycosylation includes fucosylation, sialylation, N− and O−linked glycan branching, and O−glycan truncation (25, 27). Furthermore, abnormal glycosylation also occurs in the tumor microenvironment due to hypoxia, inflammatory events, and metabolism, and it plays a crucial functional role in tumor progression and metastasis. Therefore, the glycosylation of PD-1 may not only affect the immune regulatory roles of the MAbs targeting PD-1 in T cells but may also interfere with the tumor regulatory roles of the MAbs when binding to PD-1 in tumor cells. The PD-1-specific blocking MAbs camrelizumab, MW11-h317, mAb059c, and STM418 contact the N58 glycan when binding to PD-1 (10, 17, 28, 29). Although clinical evidence supports improved overall survival rates across multiple cancer types with camrelizumab, unexpected binding of camrelizumab to VEGFR2 has been reported and may correlate with the side effects of capillary hemangiomas usually observed in clinical studies with camrelizumab (30). Structural analysis reveals that camrelizumab binds to the core region of the N-glycan of PD-1, which is conserved in the N-glycosylation of some proteins. Therefore, the binding of these MAbs to the conserved N-glycan on PD-1 may reduce the binding specificity, although further systemic investigations should be performed to evaluate the binding specificities of the MAbs that engage with the N58 glycan. Both the binding affinities and PD-1/PD-L1 blocking efficiencies of cemiplimab to N58 glycan-deficient PD-1 were similar to that of camrelizumab, as revealed in the present study. Structural analysis and sequence alignment also indicate that cemiplimab binds to N58 glycan chains with conserved HCDR2 regions similar to camrelizumab and MW11-h317. However, clinical studies for cemiplimab do not report a high prevalence of capillary hemangiomas as observed for camrelizumab (58.6%) (31). This indicates that although the conserved N58 glycan promotes the binding of these two MAbs to PD-1 in a similar mode, the binding specificities of the MAbs may vary due to the variable regions responsible for the binding to residues in PD-1.

Taken together, we report the molecular basis of cemiplimab binding to PD-1. Cemiplimab mainly utilizes its heavy chain to bind to the binding “hotspot” for therapeutic MAbs targeting PD-1, i.e., the FG loop of PD-1. Cemiplimab binds to PD-1 in a similar mode to camrelizumab, and the N58 glycan on the BC loop of PD-1 was verified to promote both the binding and blocking of cemiplimab. All of these findings facilitate our understanding of the interaction between cemiplimab and PD-1 and will benefit the future design of agents targeting glycosylated PD-1.

## Materials and Methods

### Plasmid Construction and Protein Purification

For *E. coli* cell expression, the extracellular domain of PD-1 (UniProt: Q15116, residues L25-R147) and cemiplimab-scFv [designed as a format of VL-GGGGS (4)-VH] were cloned into Novagen’s prokaryotic expression vector pET-21a(+). The two plasmids above were transformed into *E. coli* strain BL21 (DE3) pLysS cells and overexpressed as inclusion bodies under IPTG (1 mM) induction, which were verified by sodium dodecyl sulfate–polyacrylamide gel electrophoresis (SDS-PAGE). The inclusion bodies were then dissolved by dissolution buffer [6 M Gua-HCl, 10% v/v glycerol, 50 mM Tris-HCl, 100 mM NaCl, 10 mM ethylenediaminetetraacetic acid (EDTA), pH 8.0] and co-refolded as previously described (32–34). Briefly, a solution of PD-1 and cemiplimab-scFv was mixed in 1:1 molar ratio, and then, 5 ml of the mixture (30 mg/ml) added drop by drop to 2.5 L refolding buffer (100 mM Tris–HCl, 400 mM L-Arg-HCl, 2 mM EDTA, 5 mM glutathione (GSH), and 0.5 mM oxidized glutathione (GSSG), pH 8.0]. After gently stirring for 8 h, the solution was concentrated and exchanged to protein buffer (20 mM Tris–HCl, 150 mM NaCl, pH 8.0). Subsequentially, the cemiplimab-scFv/PD-1 complex protein was purified *via* size exclusion using an AKTA Pure system with Superdex™ 200 Increase 10/300 GL column.

For mammalian cell expression, the extracellular domains of PD-1 (residues L25-R147) and PD-1 N58A residue substitution mutant gene (obtained by site-directed mutagenesis) were cloned into an expression vector pCAGGS with signal peptide at the N-terminal and six histidines at the C-terminal, named as PD-1-WT and PD-1-N58A, respectively. The full-length heavy- and light-chain genes of cemiplimab and camrelizumab were cloned into the pCAGGS vector individually with *Eco*RI and *Xho*I sites, named as cemiplimab-Fc and camrelizumab-Fc. Plasmids were transiently transfected into 293F cells and incubated at 37°C for 72 h. The culture was centrifuged, and supernatant was then collected and filtered with a 0.22-μm filter. The PD-1-WT or PD-1-N58A proteins were purified first by His-Trap HP column (GE Healthcare) followed by Superdex™ 200 10/300 GL (GE Healthcare). The proteins of full-length cemiplimab and camrelizumab were purified with protein A column (GE Healthcare) before loading on a Superdex™ 200 10/300 GL (GE Healthcare, Chicago, United States). The purified protein was stored in the protein buffer (20 mM Tris–HCl, 150 mM NaCl, pH8.0). The protein purity was assessed by SDS-PAGE, and proteins were stained with Coomassie brilliant blue (**Supplementary Figure S4**). The human PD-L1 gene (full length) was cloned into (Clontech’s, Beijing, China) pEGFP-N1 vector, which was named pEGFP-PD-L1.

### Data Collection and Structure Determination

For crystal screening, 100 μl of crystallization solution is added to the reservoir of the crystallization chamber. One microliter of cemiplimab-scFv/PD-1 complex protein at a concentration of 5 mg/ml and 1 μl of the crystallization solution are pipetted onto the sitting drop post that is located at the center of beside chamber. Crystallization plates were sealed and placed at 4 or 18°C to perform a sitting drop vapor diffusion experiment. Crystals of cemiplimab-scFv/PD-1 complex were grown in 0.1 M sodium acetate, pH 5.0, 5% w/v γ-PGA (Na+ form, LM), and 20% w/v PEG 2000 MME. The diffraction data were collected at 100 K on the beamlines BL19U1 of the Shanghai Synchrotron Radiation Facility (SSRF). The collected intensities were processed and scaled using the HKL2000 software package (HKL Research). The structures were determined using molecular replacement with the program Phaser MR in CCP4 (35). The search model used in this complex was from Protein Data Bank (PDB) codes 5GGU and 6KTR with the most similar sequences. Model building was performed using COOT by hand (36). Structure refinement was done by using Phenix (37). Structure-related figures in this article were generated using PyMOL (http://www.pymol.org/). The buried surface between MAbs and PD-1 was calculated on the web server (https://www.molnac.unisa.it/BioTools/cocomaps/index.psp).

### SPR Analysis

The SPR measurements between different forms of PD-1 and MAbs were performed on the BIAcore8000 system (GE Healthcare) with Sensor Chip CM5 (GE Healthcare) at room temperature. To measure the binding characteristics between PD-1 antibodies (cemiplimab or camrelizumab) and different forms of PD-1 proteins (PD-1-WT, PD-1-N58A, and PD-1-*E. coli*), cemiplimab-scFv and camrelizumab-scFv were individually immobilized on the CM5 chip to 695 and 569 response units, respectively. Then, serially diluted PD-1-WT samples and blank control, prepared as 0 , 6.26 , 12.5, 25, 50, and 100 nM, were flowed over Sensor Chip CM5. After regeneration, PD-1-N58A protein, expressed by 293F cells, was flowed over the CM5 sensor chip with various concentrations (50–800 nM, five gradients, twofold dilution). Similarly, different concentrations of PD-1-*E. coli* (0, 0.5, 1, 2, 4, and 8 μM) were flowed over the CM5 chip. The binding kinetics were all analyzed with the Biacore™ insight evaluation software (GE Healthcare) using a 1:1 Langmuir binding model.

### FACS Analysis of PD-1/PD-L1 Blockade Assay

The pEGFP-PD-L1 plasmid was transfected into human embryonic kidney 293 cells (293T) with polyethyleneimine transfection reagent. After 24 h, cell density was adjusted to 1 × 10^7^ cells/ml with phosphate-buffered saline (PBS). PD-1-WT or PD-1-N58A protein (2 ug/ml) was respectively preincubated with different concentrations (0, 2, 4, 10, 20, 40, and 80 μg/ml) of full-length cemiplimab or camrelizumab at room temperature for 30 min. Subsequently, the 293T cells expressing PD-L1 fused enhanced green fluorescent protein (EGFP) were incubated with these mixed samples for a further 30 min at room temperature. The 293T cells were washed three times with PBS and stained with secondary APC mouse anti-His antibody (Cat: 130-119-782; clone: GG11-8F3.5.1; Miltenyi Biotec, Beijing, China) for 30 min, then washed twice with PBS, and resuspended with 300 μl of PBS for flow cytometry (BD FACS Canto Flow Cytometer, Franklin Lakes, USA). The FACS files were analyzed by FlowJo 7.6.

## Data Availability Statement

Atomic coordinates have been deposited in the Protein Data Bank (PDB, http://www.rcsb.org) under the accession code 7WVM.

## Author Contributions

GFG, ST, YC, DL, ZX, and DZ contributed to the conception and design of the study. DL, ZX, DZ, MJ, and JH performed the experiment. DL, KL, ST, and YC performed the statistical analysis. DM, XM, and GFG helped perform the analysis with constructive discussions. ST, DL, and YC wrote the first draft of the manuscript. All authors contributed to the article and approved the submitted version.

## Funding

This work was supported by the National Natural Science Foundation of China (NSFC, 31830097 and 32100752).

## Conflict of Interest

The authors declare that the research was conducted in the absence of any commercial or financial relationships that could be construed as a potential conflict of interest.

## Publisher’s Note

All claims expressed in this article are solely those of the authors and do not necessarily represent those of their affiliated organizations, or those of the publisher, the editors and the reviewers. Any product that may be evaluated in this article, or claim that may be made by its manufacturer, is not guaranteed or endorsed by the publisher.
