# Supplementary material for: PD-1 N58-Glycosylation-Dependent Binding of Monoclonal Antibody Cemiplimab for Immune Checkpoint Therapy
Source: Front Immunol. 2022 Mar 2;13:826045. doi: 10.3389/fimmu.2022.826045 (PMC8924070; doi:10.3389/fimmu.2022.826045)
Supplement: Supplementary file 1 [file DataSheet_1.docx]

**
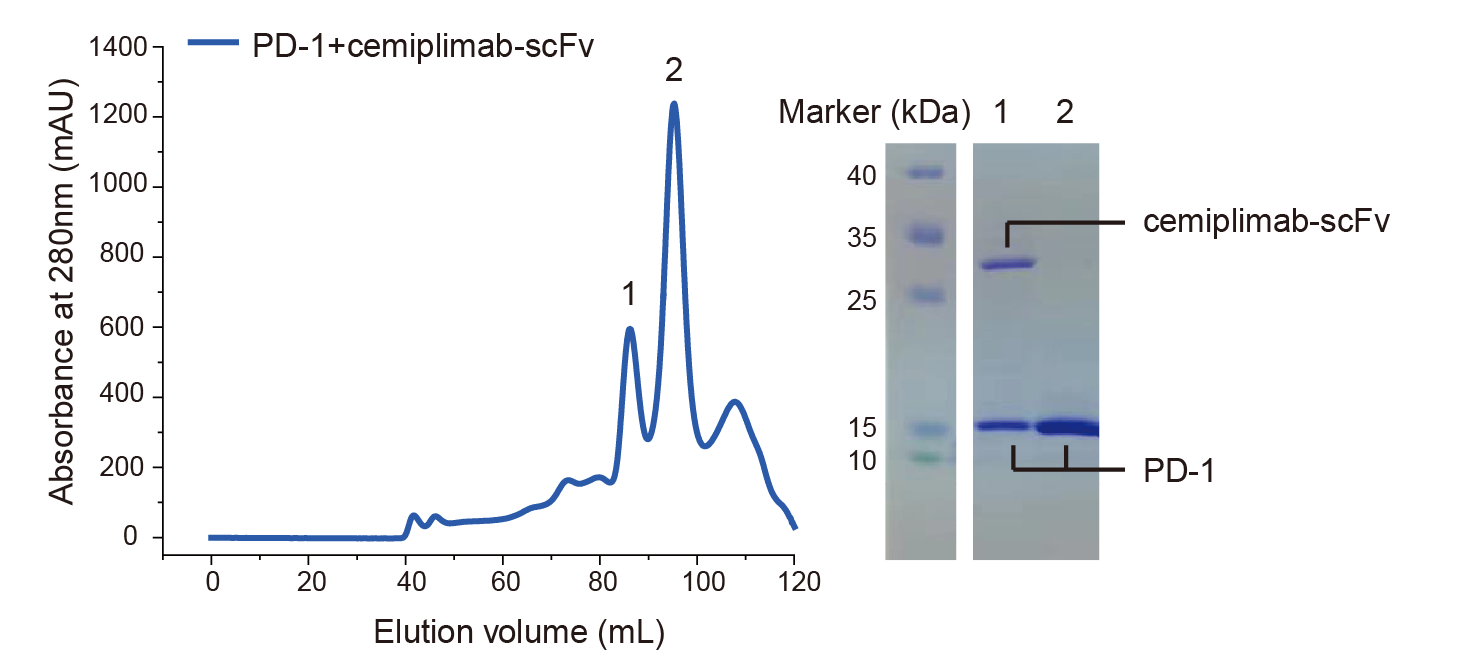
** **Supplementary Figure 1.** The cemiplimab-scFv/PD-1 complex purified by gel filtration column.

Gel filtration profiles of cemiplimab/PD-1 (blue) are analyzed by size-exclusion chromatography as indicated. The SDS–PAGE analyses are shown with peak 1 of cemiplimab/PD-1 complex, and peak 2 of PD-1.

**
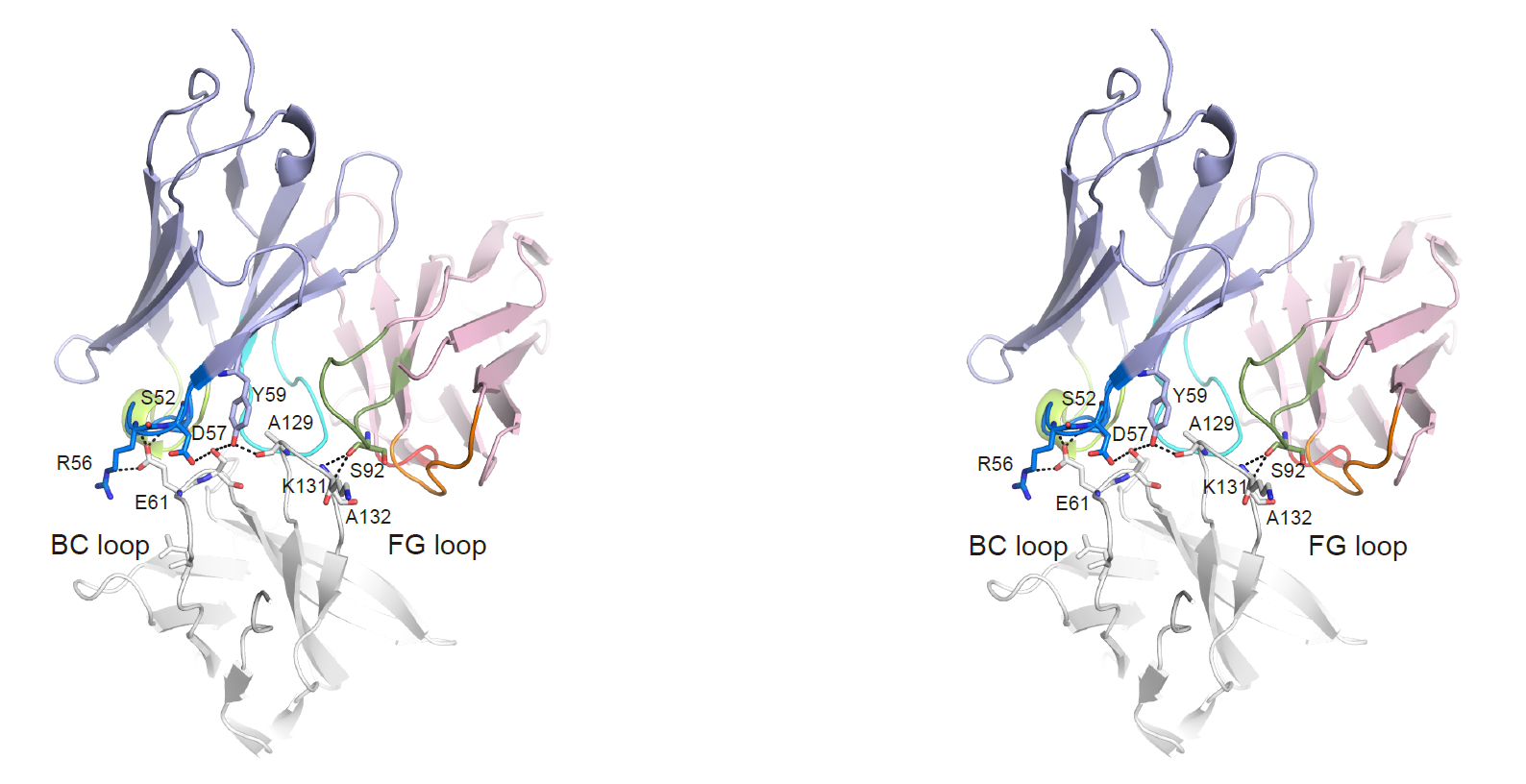
**

**Supplementary Figure 2.** A cross-eye stereo view (1.0 σ contour level) of the binding details between cemiplimab and PD-1.

The detailed binding of cemiplimab to the FG loop and BC loop of PD-1. The residues taking part in forming hydrogen bonds are shown as sticks. The hydrogen bonds between residues are shown as a dashed line in black.


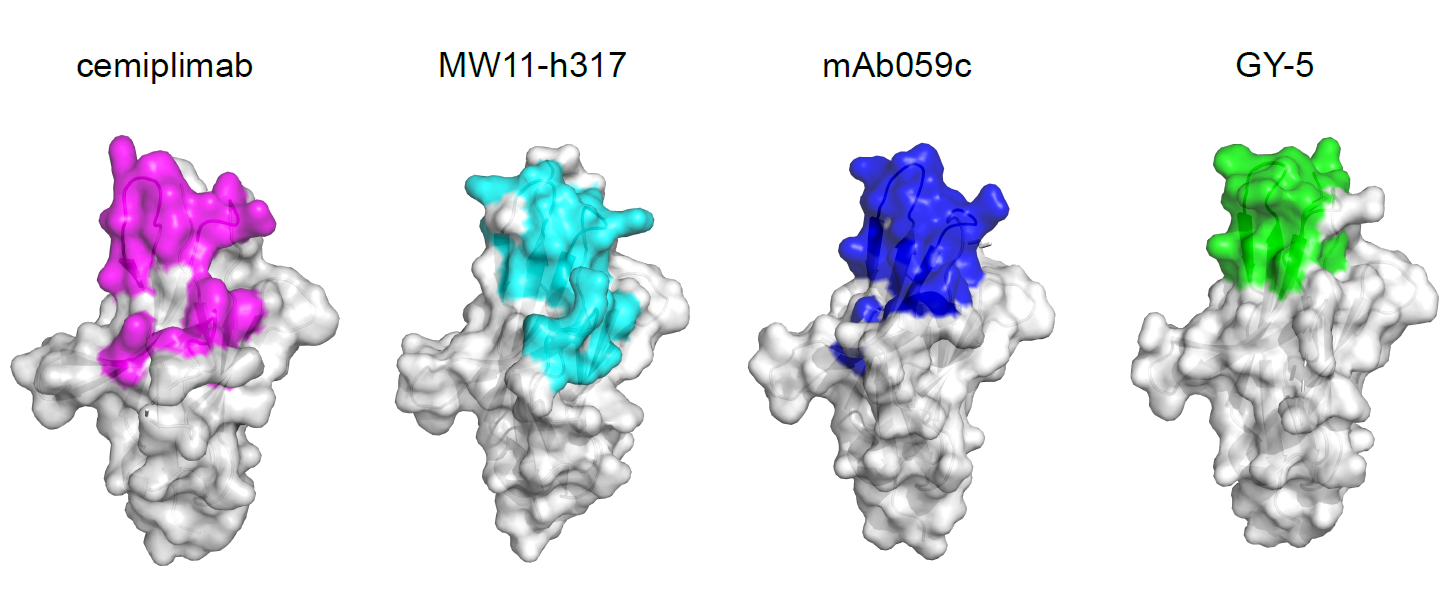
 **Supplementary Figure 3.** Comparative binding of PD-1 and MAbs.

The binding area of the MAbs targeting PD-1(PDB code: 4ZQK), cemiplimab, MW11-h317 (PDB code: 6JJP), mAb059c (PDB code: 6K0Y) and GY-5 (PDB code: 6J15) are colored in magenta, cyan, blue and green respectively.


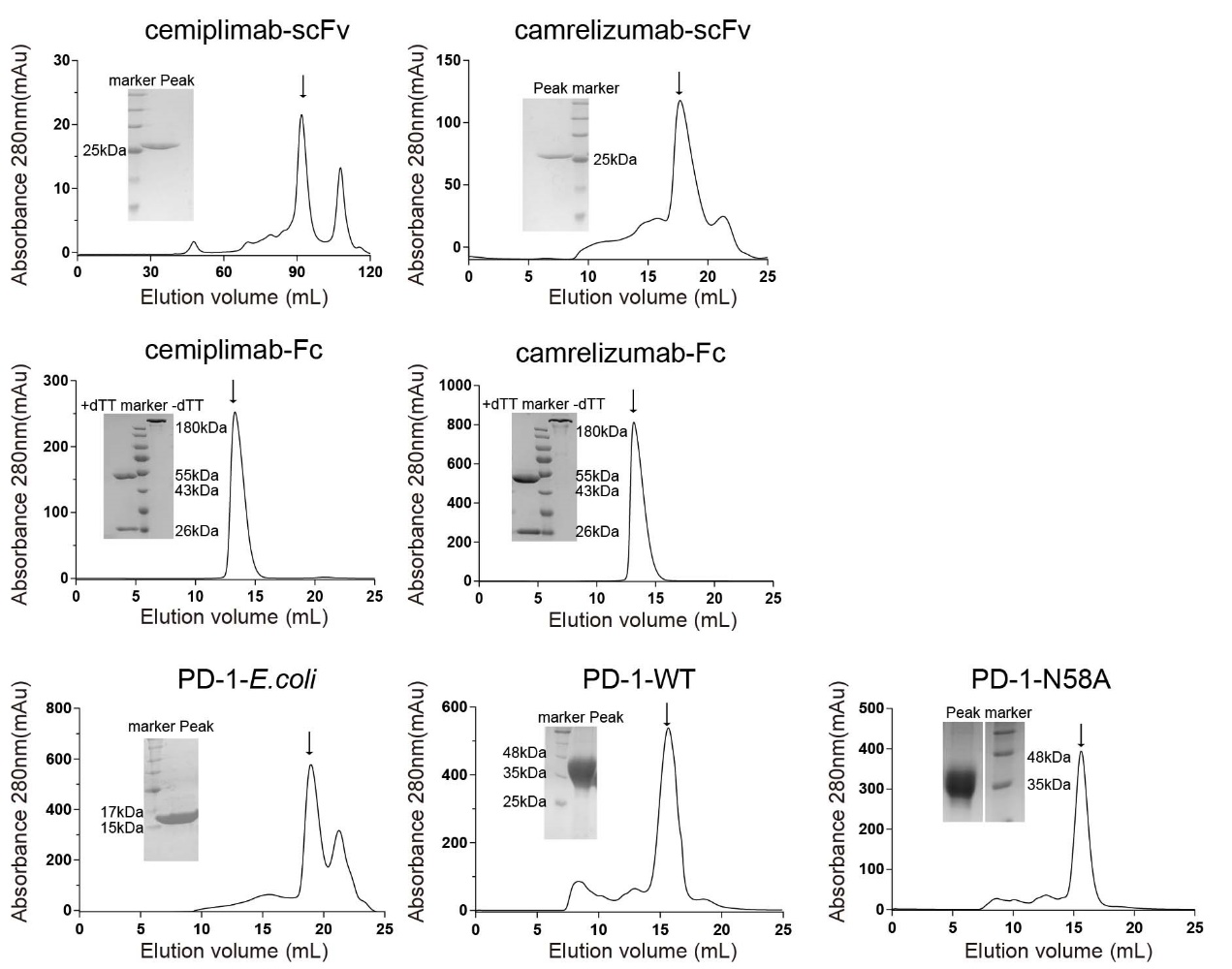


**Supplementary Figure 4.** Different forms of PD-1 and anti-PD-1 antibodies used in SPR or FACS analysis purified by gel filtration column.

The peaks marked by arrows are the target proteins, and protein purity was assessed by SDS-PAGE.

**Supplementary Table 1.** SPR analysis of the binding between MAbs and different forms of PD-1.

|  | K_a_ (Ms) | K_d_ (1/s) | K_D_ (nM) |
| --- | --- | --- | --- |
| cemiplimab vs PD-1-WT | 1.19 ± 0.19×10^5^ | 1.98 ± 0.06×10^4^ | 1.68 ± 0.23 |
| cemiplimab vs PD-1-N58A | 2.61 ± 0.30×10^5^ | 2.74 ± 0.03×10^-2^ | 106.0 ± 10.76 |
| cemiplimab vs PD-1-*E. coli* | 6.87 ± 0.72×10^4^ | 4.70 ± 0.36×10^-2^ | 691.38 ± 117.72 |
| camrelizumab vs PD-1-WT | 2.28 ± 0.20×10^5^ | 1.09 ± 0.06×10^3^ | 4.80 ± 0.19 |
| camrelizumab vs PD-1-N58A | 3.48 ± 0.27×10^5^ | 1.71 ± 0.08×10^-1^ | 492.85 ± 26.06 |
| camrelizumab vs PD-1-*E. coli* | 1.18 ± 0.07×10^5^ | 3.08 ± 0.01×10^-1^ | 2630 ± 109.13 |
